# The Hospital System of India

**Published:** 1915-10-09

**Authors:** 


### THE HOSPITALS OF INDIA AND DUBLIN.

### The Hospital System of India.

The recently published Annual Report on the progress and condition of India contains some interesting particulars concerning the hospitals of India. These are divided into three groups—namely, (1) civil hospitals and dispensaries, (2) State special and railway hospitals, and (3) private non-aided institutions. The statistics, which are somewhat belated, deal with the year 1913, and show that during that year there were 2,820 institutions in the first group, and that the total number of in-patients was 515,062, and of out-patients over thirty millions. The number of institutions in the second category was 351, the in-patients treated numbering 98,171, and the out-patients 2,331,969. In the third group the number of institutions was 697, the number of in-patients 57,252, and the number of out-patients 4,828,357. During the year in question an electrical annex was added to the Medical College Hospital in Calcutta, and it has been decided to build a new eye hospital. A medical school is being established at Nagpur, the new hospital in connection with King George's Medical College, Lucknow, was opened, and in Bombay the equipment of civil hospitals is being brought up to date. In Bengal a Medical Registration Act was passed, and similar Acts have since been passed in other provinces. The system of exacting fees from well-to-do persons who use hospitals is being tried in Bengal, the Central Provinces, and the Punjab. From Assam, Behar and Orissa, the Central Provinces and Burma, a dearth of sub-assistant surgeons is reported, despite improvements in pay. Many provinces are paying attention to increasing the supply of women doctors, and in the Punjab the Ludhiana School of Medicine for Women is being assisted by a Government grant. There are five medical colleges (Bombay, Madras, Calcutta, Lahore, and Lucknow), the students in which numbered 2,080, including 101 women. There are also fifteen medical schools, the students in which numbered 2,126. There is an *x*-ray institution at Dehra Dun, where two classes of instruction were attended in all by thirty-seven students. A branch installation has been opened at Delhi and another has been approved for Simla. There were Pasteur Institutes for anti-rabic treatment at Kasauli (Punjab) and Coonoor (Madras), and the establishment of a Pasteur Institute at Rangoon has been sanctioned. The treatment of lunatics at asylums prevails on only a small scale in India, where insanity is less prevalent than in European countries. There are many leper asylums, and many asylums or homes, frequently under some sort of Government supervision, including about fifty asylums of the Mission to Lepers.

